# The Chinese Version of the Breast Cancer Literacy Assessment Tool: Translation, Adaptation, and Validation Study

**DOI:** 10.2196/43002

**Published:** 2023-04-19

**Authors:** Yi Shan, Meng Ji

**Affiliations:** 1 School of Foreign Studies Nantong University Nantong China; 2 School of Languages and Cultures The University of Sydney Sydney Australia

**Keywords:** breast cancer literacy assessment tool, translation, adaptation, psychometric evaluation, validity, reliability, breast cancer, cancer, tool, women, prevention, detection, intervention, assessment, education, breast cancer literacy, assessment tool, cancer awareness, health literacy

## Abstract

**Background:**

Breast cancer is the most common cancer among Chinese women, with an age-standardized prevalence of 21.6 cases per 100,000 women. Limited cancer health literacy reduces females’ ability to engage in cancer prevention and detection. It is necessary to assess Chinese women’s breast cancer literacy to deliver targeted interventions and effective education. However, there is no Breast Cancer Literacy Assessment Tool (B-CLAT) available in China currently.

**Objective:**

This study aimed to translate and linguistically and culturally adapt the B-CLAT into a simplified-Chinese version (C-B-CLAT), and then validate its psychometric properties by administering it to Chinese college students.

**Methods:**

First, we translated the B-CLAT into a simplified-Chinese version and verified its validity and reliability using rigorous translation and validation guidelines proposed in previous studies. We then evaluated the psychometric properties among 50 female participants with a mean age of 19.62 (SD 1.31) years recruited from Nantong University, China.

**Results:**

Items 1, 6, 8, 9, 10, 16, 17, 20, 21, 22, 23, 24, 25, 26, 29, and 30 were deleted to increase the relevant subscale internal consistency. Items 3, 12, 13, 14, 18, 20, 27, and 31 were deleted due to their Cronbach *α* being lower than .5 in the test-retest analysis. After deletion, the internal consistency of the entire scale was fair with *α*=.607. The prevention and control subscale had the highest internal consistency with *α*=.730, followed by the screening and knowledge subscale with *α*=.509, while the awareness subscale had the lowest internal consistency with *α*=.224. The intraclass correlation coefficient for the C-B-CLAT (items 2, 4, 5, 7, 11, 15, 28, 32, 33, and 34) was fair to excellent (odds ratio [OR] 0.88, 95% CI 0.503-0.808). The values of Cronbach *α* for items 2, 4, 5, 7, 11, 15, 28, 32, 33, and 34 ranged from .499 to .806, and the *α* value for the C-B-CLAT was .607. This indicates fair test-retest reliability. The mean difference in the C-B-CLAT scores between stage 1 and stage 2 was 0.47 (OR 0.67, 95% CI −0.53 to 1.47), which was not significantly different from zero (t_48_=0.945; *P*=.35). This result implies that the C-B-CLAT produced the same scores at stage 1 and stage 2 on average, thus showing good agreement in the C-B-CLAT scores between stage 1 and stage 2. The SD of the difference was 3.48. The 95% limits of agreement were −6.34 to 7.28.

**Conclusions:**

We developed a simplified-Chinese version of the B-CLAT through translation and adaptation. Psychometric properties testing has proven this version valid and reliable for assessing breast cancer literacy among Chinese college students.

## Introduction

### Background

Breast cancer is the most prevalent cancer among women globally, constituting around 23% of all female cancers [1,2]. It is expected that over 2 million women will be diagnosed with cancer by 2030 with increasing percentages from low-income countries [3]. Breast cancer is also the most common cancer among Chinese women with an age-standardized prevalence of 21.6 cases per 100,000 women [4]. Newly diagnosed breast cancer cases in China represent 12.2% of all newly diagnosed breast cancer cases worldwide, and deaths from breast cancer in China constitute 9.6% of all cancer-induced deaths worldwide [5]. There is a rapid increase in China’s proportional contribution to the global cancer rate due to the improving socioeconomic status and unique reproductive patterns of Chinese populations [5].

Health literacy is a significant determinant of health outcomes [6]. It has been associated with improved compliance with health care recommendations, health appointments, and the adoption of preventive care (eg, cancer screening) [7]. Limited health literacy was found to effectively predict inadequate use of health care resources and poor health outcomes among susceptible populations [8,9]. Breast and cervical cancer literacy have been defined as “a woman’s functional understanding of her personal and familial risk of the disease, including how to minimize her risk and the risk of her family through preventive early detection screenings, lifestyle changes, and understanding how to access the health system and engage providers to minimize her risk and the risk of her family” [10]. Limited cancer health literacy reduces females’ ability to engage in cancer prevention and detection [10]. Given the global prevalence of female breast cancer, it is necessary to assess breast cancer literacy among women worldwide to deliver more targeted interventions and conduct more effective education. To this end, some Breast Cancer Literacy Assessment Tools (B-CLAT) have been well-developed and validated. Williams et al [10] produced the first version of the 16-item B-CLAT as a disease-specific literacy scale. This 16-item B-CLAT was verified in feasibility studies and culturally and linguistically translated [11]. The second version of the B-CLAT was designed to measure functional breast cancer literacy [12]. Williams et al [13] created an assessment instrument measuring women’s understanding of their personal and familial risks of breast and cervical cancers. Han et al [6] made the Assessment of Health Literacy in Cancer Screening to measure the effect of a health literacy–focused intervention to promote breast and cervical cancer screening. These tools, among many others, can provide important clinical implications in that informing physicians and pharmacists early on in health care delivery can allow for timely interventions designed to reduce adverse health outcomes for patients with limited cancer health literacy [14].

Breast cancer incidence has grown by 20%-30% in the past 3 decades, and it annually increases by 3%-5% in China [15,16]. This yearly rise is considerably higher than the global average growth of 1.5% [17,18]. It is therefore imperative to assess cancer health literacy among Chinese women to deliver timely, tailored education about and interventions in female breast cancer prevention and treatment. However, there is no breast cancer literacy assessment measure currently available in China to the best of our knowledge. In this case, measures of literacy are urgently needed to allow for assessing people’s literacy competence and recommending promising interventions and strategies [8]. Considering that “A gap exists for linguistically and culturally sensitive measurements of functional breast cancer literacy that adheres to psychometric rigors” [12], we need to develop a Chinese-language breast cancer literacy assessment scale that is linguistically and culturally appropriate to the Chinese social-cultural context.

In the context of the lack of an instrument, translating validated scales for use in different language studies is regarded as a rapid and practical approach to assessment [19] for developing new tools that entail painstaking efforts and substantial time and cost investments [20]. It is, accordingly, imperative to translate quantitative tools into the language of the culture that is investigated for studies in which these instruments are used [21]. As such, we intended to translate the B-CLAT into a Chinese version. As Mohamad Marzuki and colleagues [22] strongly suggested, established, available, and reliable instruments need to be adapted, tested, and documented cross-linguistically. It is also essential to adapt tools cross-culturally [23]. We would, therefore, adapt the Chinese version of the B-CLAT to Chinese culture and evaluate the psychometric properties of the adapted tool to make sure of its applicability and efficacy. Research tools must be proven reliable in each culture that is studied to explore the health care needs of individuals from diverse cultural backgrounds [24]. A reliable translated assessment scale needs to undergo rigorous translation and appropriate adaptation to ensure semantic and content equivalence in cross-cultural studies [19,24,25]. Informed by these studies, we would translate and adapt the B-CLAT and evaluate the psychometric properties of the translated and adapted version rigorously in this study.

### Objective

This study aimed to translate and linguistically and culturally adapt the B-CLAT into a simplified-Chinese version, and then evaluate its convergent validity and psychometric properties by administering it to Chinese college students.

## Methods

### Overall Design

Informed by Wångdahl et al [26], we conducted the prospective psychometric evaluation study of the Chinese version of the B-CLAT in two phases: (1) translation and content validation and (2) psychometric evaluation. The B-CLAT was designed to specifically measure functional health literacy specific to breast cancer for the benefit of cancer researchers and health educators [12]. This scale consists of 34 items in multiple-choice and true or false format allocated into three domains: (1) cancer awareness (6 items), (2) knowledge of screening modalities (13 items), and (3) prevention and control (15 items). It has been rigorously tested.

### Phase 1: Translation and Content Validation

#### Overview

**Figure 1:**
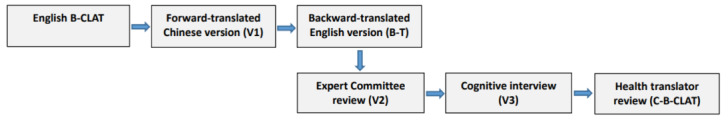
Process of developing the C-B-CLAT. This figure was adapted from Barros et al [27] which is published under Creative Commons Attribution 4.0 International License [38]. B-CLAT: Breast Cancer Literacy Assessment Tool; C-B-CLAT: simplified-Chinese version of the B-CLAT.

Following rigorous translation and validation guidelines proposed in previous studies [27-37], we translated and adapted the B-CLAT into a simplified-Chinese version and verified the validity of the newly developed tool. Figure 1 presents the development of the simplified-Chinese version of the B-CLAT (C-B-CLAT) through forward translation, backward translation, cognitive interview, and expert review.

#### Forward and Backward, Initial Translation by 2 Native Chinese Speakers

To adopt and apply the B-CLAT in a different sociocultural background, we needed to go through a rigorous process of language translation and cultural adaptation [27,28]. Informed by Wild et al [29], we forward-translated and linguistically and culturally adapted the English B-CLAT into a simplified Chinese version (V1). A native Chinese translator fluent in English made the forward translation. Cross-cultural translation frequently involves problems concerning the translation quality and comparability of research results in diverse cultural and ethnic communities [30]. It is particularly challenging to adapt an instrument into a culturally relevant and comprehensible version while maintaining its intended meanings and purposes because of the cross-cultural variations in the values and meanings of the composing constructs of the instrument [30]. Considering these challenges, we attached great importance to functional equivalence to ensure cross-cultural validity in forward translation.

Following the proposal of Sperber [30], another native Chinese bilingual translator with rich experience in health and medical material translation back-translated V1 into an English version (B-T). This bilingual translator was blind to the original English version of the B-CLAT to ensure the quality of the back translation [31]. Through back translation, we intended to find gaps between the source and target versions and thus revise concepts and expressions that were not functionally equivalent [30]. Back translation may be repeated until functional equivalence was achieved. Through back translation, we can validate the adequacy of instrument translation [30].

#### Expert Committee Review of the Preliminary Translation

The expert committee comprised all authors of this study, including 2 native Chinese bilingual translators and 3 health professionals. This committee reviewed the B-T and the original B-CLAT to ascertain whether semantic, idiomatic, experiential, and conceptual equivalence was secured between the 2 English versions [33,34]. We ensure the linguistic and scientific accuracy and comprehensibility of the preliminary translation by resolving all discrepancies at the panel meeting of the expert committee. As a result, we produced the reviewed Chinese version (V2).

#### Cognitive Interview Following a “Think-Aloud Protocol”

In total, 10 volunteers were recruited for a cognitive interview, including 5 women and 5 men with year 6, year 9, year 12, and university education who were aged 32 to 66 years. Based on the “think-aloud protocol” [35], they were asked to fill out V2 while “thinking aloud,” to provide open feedback on whether and how they understood the questionnaire [36]. This step aimed to test the understandability of V2. Subsequently, the 10 volunteers and all authors of this study resolved problems with the question organization, the instrument layout (including the font size), and elusive questions or concepts [27]. We focused on challenging questions and concepts related primarily to cultural relevance (whether they were relevant to the participants’ daily life) and linguistic accessibility (whether they were comprehensible or ambiguous to the participants). This step guaranteed the face validity of the translated tool, resulting in V3.

#### Review by Professional Health Translators With Extensive Community Engagement Experiences

Two professional health translators with extensive community engagement experience reviewed V3 to ensure its comprehensibility and cultural acceptability. In this process, what was used as a review criterion was the suitability assessment of materials for the evaluation of health information in readability and understandability proposed by Doak et al [37]. This step finalized the translated and adapted version (C-B-CLAT).

### Phase 2: Psychometric Evaluation

#### Participant Recruitment and Questionnaire Survey

Participants were recruited from college students of Nantong University, China through randomized sampling. The inclusion criteria were as follows: (1) being 18 years or older, (2) understanding the questions in the questionnaire, and (3) participating in the survey voluntarily. The web-based survey was divided into 2 stages: August 8, 2022, to August 14, 2022 (stage 1) and September 3, 2022, to September 9, 2022 (stage 2). Those who had participated in stage 1 were invited to participate in stage 2 for the test-retest purpose. This interval of over 2 weeks between the test and the retest could avoid biased answers in retest caused by the eventual recall of questions or previous answers [23]. Participants received written information on this study, including the study objective and steps, voluntary participation, and an option of withdrawal during any phase. They were assured confidentiality and secure data storage. The C-B-CLAT complete with age, gender, and education was administered via *wenjuanxing* [39], the most popular internet survey platform in China.

#### Data Collection and Analysis

The sample size was up to the actual number of students’ responses to the web-based questionnaire. The data were collected on August 15, 2022 (stage 1) and September 10, 2022 (Stage 2), respectively, and stored in an Excel file. A valid questionnaire must have all question items answered according to our predefined data inclusion criterion. All valid data were subjected to psychometric evaluation using SPSS version 22.0 (IBM Corp).

#### Psychometric Testing

##### Internal Consistency

We used internal consistency that was measured using Cronbach *α* to assess the degree of interrelatedness among items on a test [40]. *α* varies between 0 and 1, and higher α values imply higher degrees to which items on a test are interrelated [40]. *α* values from .70 to .95 are considered good, and *α* values from .60 to .69 are considered fair [40]. α is influenced by the number of items on a test; the more items on a test, the higher the *α* values are [40]. We measured the internal consistency of the entire C-B-CLAT and its subscales.

##### Test-Retest Reliability

Test-retest reliability of the C-B-CLAT was assessed by calculating the intraclass correlation coefficient (ICC) using a 2-way random effects model, which assumes that random errors come both from raters and participants [41]. The ICC is determined through (between subjects variability) ÷ (between subjects variability + error); as the error term decreases, the ICC varies from 0 to 1 indicating perfect reliability [41]. Fleiss (as cited in Oremus et al [42]) proposed a classification for the strength of test-retest reliability based on the ICC as follows: <0.40 poor, 0.40-0.75 fair to good, and >0.75 excellent.

##### Bland-Altman Method

The Bland-Altman method was used to determine the agreement between the C-B-CLAT scores at stage 1 and stage 2. This method plots the agreement between 2 quantitative measurements and quantifies this agreement by constructing agreement limits, which are measured using the mean and SD of the differences between the 2 measurements: the upper agreement limit = 1.96 * SD + mean score and the lower agreement limit = 1.96 * SD − mean score [43,44]. The x-axis represents the mean of the paired measurements, whereas the y-axis of the Bland-Altman plot represents the difference between paired measurements [43,44]. All data points should lie within ±2 SDs of the mean difference [43,44].

### Ethical Considerations

Ethical approval was obtained from the Students’ Affairs Department and the Humanities and Social Sciences Department at Nantong University. It is an official practice in this university to ask the Students’ Affairs Department and Humanities and Social Sciences Department for approval before collecting data from students. We followed this practice. Besides, there is no ethics review board at Nantong University. Therefore, a review number or code for this study could not be provided. Written consent was obtained from participants in the study. Study data were anonymous or deidentified for privacy and confidentiality protection. We recruited students who were willing to support our research without compensation.

## Results

### Descriptive Statistics of Participant Demographics

Table 1 presents the results of a descriptive analysis of the data collected. A total of 50 responses were collected from 50 female participants via *wenjuanxing*. Their age ranged from 17 to 22 years (mean 19.62, SD 1.31 years). They were in 4 different grades with an average of 2.24 (SD 1.10) years of college education: freshman (n=16, 32%), sophomore (n=15, 30%), junior (n=10, 20%), and senior (n=9, 18%). None of them reported an experience of having a breast disease.

**Table 1:** Descriptive statistics.

	Minimum	Maximum	Mean (SD)
B-CLAT1^a^	1.00	2.00	1.84 (0.37)
B-CLAT2	2.00	3.00	2.16 (0.37)
B-CLAT3	2.00	2.00	2.00 (0.00)
B-CLAT4	1.00	5.00	3.54 (1.58)
B-CLAT5	1.00	2.00	1.82 (0.39)
B-CLAT6	1.00	2.00	1.98 (0.14)
B-CLAT7	1.00	3.00	1.50 (0.79)
B-CLAT8	1.00	4.00	1.82 (0.75)
B-CLAT9	1.00	3.00	1.98 (0.51)
B-CLAT10	1.00	2.00	1.08 (0.27)
B-CLAT11	1.00	3.00	2.18 (0.52)
B-CLAT12	1.00	2.00	1.16 (0.37)
B-CLAT13	1.00	3.00	2.88 (0.44)
B-CLAT14	1.00	3.00	1.04 (0.28)
B-CLAT15	1.00	5.00	3.10 (1.82)
B-CLAT16	1.00	5.00	3.54 (1.68)
B-CLAT17	1.00	2.00	1.94 (0.24)
B-CLAT18	1.00	2.00	1.92 (0.27)
B-CLAT19	1.00	2.00	1.38 (0.49)
B-CLAT20	1.00	2.00	1.48 (0.50)
B-CLAT21	1.00	2.00	1.08 (0.27)
B-CLAT22	1.00	3.00	2.52 (0.86)
B-CLAT23	1.00	2.00	1.02 (0.14)
B-CLAT24	1.00	2.00	1.34 (0.48)
B-CLAT25	1.00	2.00	1.20 (0.40)
B-CLAT26	1.00	2.00	1.12 (0.33)
B-CLAT27	1.00	2.00	1.06 (0.24)
B-CLAT28	1.00	2.00	1.88 (0.33)
B-CLAT29	1.00	5.00	4.70 (1.04)
B-CLAT30	1.00	2.00	1.52 (0.50)
B-CLAT31	1.00	2.00	1.32 (0.47)
B-CLAT32	1.00	2.00	1.76 (0.43)
B-CLAT33	1.00	2.00	1.26 (0.44)
B-CLAT34	1.00	2.00	1.80 (0.40)
Education	1.00	4.00	2.24 (1.10)
Age (years)	17.00	22.00	19.62 (1.31)
Gender	2.00	2.00	2.00 (0.00)
Breast disease history	No	2.00	No (0.00)
Valid N (listwise)	50.00	N/A^b^	N/A

^a^B-CLAT1 to B-CLAT34 refer to items 1-34 in the Breast Cancer Literacy Assessment Tool.

^b^N/A: not applicable.

### Psychometric Testing

#### Internal Consistency

Items 1, 6, 8, 9, 10, 16, 17, 20, 21, 22, 23, 24, 25, 26, 29, and 30 were deleted to increase the relevant subscale internal consistency. Items 3, 12, 13, 14, 18, 20, 27, and 31 were deleted due to their Cronbach *α* being lower than .5 in the test-retest analysis. After deletion, the internal consistency of the entire scale was fair with *α*=.607, as shown in Table 2. The prevention and control subscale had the highest internal consistency with *α*=.730, followed by the screening and knowledge subscale with *α*=.509, while the awareness subscale had the lowest internal consistency with *α*=.224, as shown in Table 2.

**Table 2:** Internal consistency analysis of subscales of the C-B-CLAT.^a^.

Scale	Scale name	Cronbach *α*	Cronbach *α* based on standardized items	Items, n	Items deleted	Items retained
1	Awareness	.224	.351	3	1,3,6	2,4,5
2	Screening and knowledge	.509	.665	3	8,9,10,12,13,14,16,17,18,19	7,11,15
3	Prevention and control	.730	.747	4	20,21,22,23,24,25,26,27,29,30,31	28,32,33,34
4	Entire scale	.607	.610	10	1,3,6,8,9,10,12,13,14,16,17,18,19,20,21,22,23,24,25,26,27,29,30,31	2,4,5,7,11,15,28,32,33,34

^a^C-B-CLAT: simplified-Chinese version of the Breast Cancer Literacy Assessment Tool.

#### Test-Retest Reliability

In total, 1 participant failed to participate in stage 2. In total, 49 participants (98%) had a test-retest interval of 19 days. The ICC for the C-B-CLAT (items 2, 4, 5, 7, 11, 15, 28, 32, 33, and 34) was fair to excellent (odds ratio [OR] 0.88, 95% CI 0.503-0.808, as presented in Table 3). The values of Cronbach *α* for items 2, 4, 5, 7, 11, 15, 28, 32, 33, and 34 ranged from .499 to .806, and the *α* value for the C-B-CLAT (items 2, 4, 5, 7, 11, 15, 28, 32, 33, and 34) was .607, as presented in Table 4. This indicates fair test-retest reliability.

**Table 3:** Test-retest reliability analysis I of retained items of the C-B-CLAT.^a^

Scale items and test	ANOVA with Friedman test					Intraclass correlation coefficient (average measures using absolute agreement; 95% CI)
	Sum of squares	*df*	Mean square	Friedman chi-square	*P* value	
B-CLAT2^b^	0.041	1.000	0.041	0.500	.48	0.607 (0.302-0.778)
B-CLAT4	0.163	1.000	0.163	0.101	.75	0.503 (0.113-0.721)
B-CLAT5	0.010	1.000	0.010	0.200	.66	0.808 (0.660-0.892)
B-CLAT7	0.653	1.000	0.653	2.286	.13	0.615 (0.325-0.782)
B-CLAT11	0.827	1.000	0.827	5.400	.02	0.620 (0.332-0.785)
B-CLAT15	1.469	1.000	1.469	0.742	.39	0.571 (0.240-0.758)
B-CLAT28	0.255	1.000	0.255	3.571	.06	0.681 (0.439-0.819)
B-CLAT32	0.092	1.000	0.092	1.286	.26	0.787 (0.624-0.879)
B-CLAT33	0.163	1.000	0.163	2.000	.16	0.702 (0.476-0.831)
B-CLAT34	0.163	1.000	0.163	2.000	.16	0.724 (0.515-0.844)

^a^C-B-CLAT: simplified-Chinese version of the B-CLAT.

^b^B-CLAT: Breast Cancer Literacy Assessment Tool.

**Table 4:** Test-retest reliability analysis II of retained items of the C-B-CLAT.^a^

Scale items and test		Scale reliability statistics			
			Cronbach *α*	Cronbach *α* based on standardized items	Mean (SD)
**B-CLAT2^b^**			.604	.604	
	Test				2.163 (0.373)
	Retest				2.122 (0.389)
**B-CLAT4**			.499	.499	
	Test				3.592 (1.553)
	Retest				3.510 (1.583)
**B-CLAT5**			.806	.806	
	Test				1.816 (0.391)
	Retest				1.796 (0.407)
**B-CLAT7**			.622	.627	
	Test				1.469 (0.767)
	Retest				1.306 (0.652)
**B-CLAT11**			.643	.643	
	Test				2.184 (0.527)
	Retest				2.000 (0.500)
**B-CLAT15**			.570	.570	
	Test				3.143 (1.814)
	Retest				3.388 (1.824)
**B-CLAT28**			.692	.706	
	Test				1.878 (0.331)
	Retest				1.776 (0.422)
**B-CLAT32**			.788	.789	
	Test				1.755 (0.434)
	Retest				1.694 (0.466)
**B-CLAT33**			.706	.710	
	Test				1.265 (0.446)
	Retest				1.184 (0.391)
**B-CLAT34**			.728	.731	
	Test				1.796 (0.407)
	Retest				1.714 (0.456)
**Total scale sum**			.607	.610	
	Test				25.9184 (3.45710)
	Retest				25.4490 (3.07586)

^a^C-B-CLAT: simplified-Chinese version of the B-CLAT.

^b^B-CLAT: Breast Cancer Literacy Assessment Tool.

#### Bland-Altman Results

**Figure 2:**
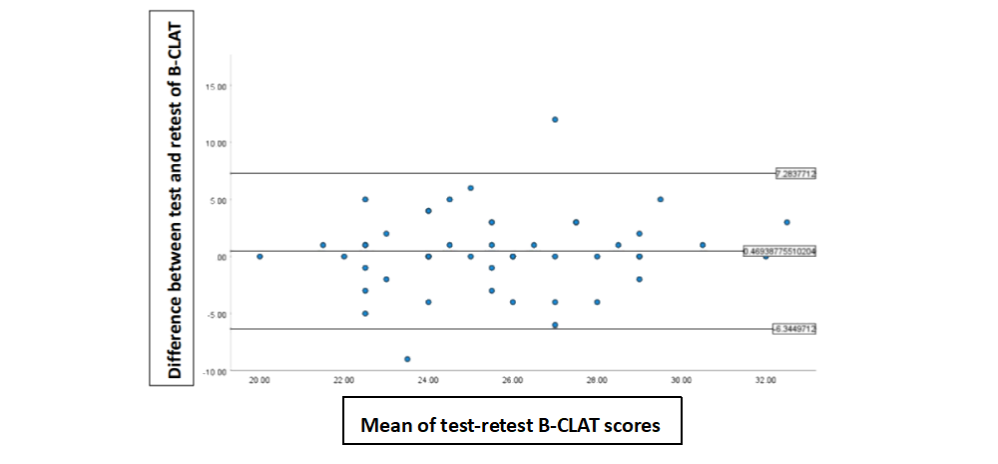
Bland-Altman plots for the simplified-Chinese version of the B-CLAT (C-B-CLAT) scores at stage 1 and stage 2. The middle line represents the mean difference between the C-B-CLAT scores between stage 1 and stage 2. The lower and upper lines represent the upper and lower 95% confidence limits, respectively. B-CLAT: Breast Cancer Literacy Assessment Tool.

The mean difference in the C-B-CLAT scores between stage 1 and stage 2 was 0.47 (OR 0.88, 95% CI −0.53 to 1.47), which was not significantly different from zero (t_48_=0.945; *P*=.35), as shown in Figure 2 and Tables 5-7. This result implies that the C-B-CLAT produced the same scores at stage 1 and stage 2 on average, thus showing good agreement in the C-B-CLAT scores between stage 1 and stage 2. The SD of the difference was 3.48, as shown in Tables 5-7. The 95% limits of agreement were −6.34 to 7.28, as shown in Figure 2.

**Table 5:** One-sample statistics of the simplified-Chinese version of the Breast Cancer Literacy Assessment Tool scores at stage 1 and stage 2 (N=49).

	Mean (SD)	SE mean
dff	0.4694 (3.47672)	0.49667

**Table 6:** One-sample test of the simplified-Chinese version of the Breast Cancer Literacy Assessment Tool scores at stage 1 and stage 2 (test value=0).

	*T* test (*df*)	Significance			Mean difference		95% CI of the difference	
		One-sided *P* value	Two-sided *P* value			Lower		Upper
dff	0.945 (48)	.18	.35	0.46939		−0.5292		1.4680

**Table 7:** One-sample effect sizes of the simplified-Chinese version of the Breast Cancer Literacy Assessment Tool scores at stage 1 and stage 2.

			Standardizer^a^	Point estimate	95% CI	
					Lower	Upper
**dff**						
		Cohen *d*^b^	3.47673	0.135	−0.147	0.416
		Hedges correction^c^	3.53225	0.135	−0.145	0.409

^a^The denominator used in estimating the effect sizes.

^b^Cohen *d* uses the sample SD.

^c^Hedges correction uses the sample SD plus a correction factor.

## Discussion

### Overview

Limited cancer health literacy prevents patients from fully benefiting from cancer treatment, causing negative health outcomes [14]. Precisely identifying patients with limited cancer health literacy remains a critical clinical challenge in the context of cancer being the leading cause of death in China [14]. To rise to this challenge, a linguistically and culturally appropriate assessment tool is needed to assess the cancer health literacy status of Chinese-speaking populations. In this context, we translated and adapted the English B-CLAT into a simplified-Chinese version and then evaluated its psychometric properties by administering it to Chinese college students. We found this newly developed scale valid and reliable for assessing breast cancer literacy among this population.

### Principal Findings

Rigorous language translation and cultural adaptation of a tool is an essential process to make it appropriate in different sociocultural settings [21,27,28,30,31]. In this process, forward translation, backward translation, cognitive interview, and expert review are indispensable to ensure the translation quality and comparability of the tool in diverse cultural and ethnic communities [21]; adapt it into a culturally relevant and comprehensible version [21]; and achieve semantic, idiomatic, experiential, and conceptual equivalence between the source and target versions [30,33,34]. Meanwhile, the readability and understandability of a newly developed instrument need to be assessed to guarantee its suitability for the evaluation of health information [37]. As such, it is a challenging task to translate and adapt an instrument cross-culturally [21,30].

Our research results are somehow consistent with those reported in the validation of the original B-CLAT [12,13,45]. The C-B-CLAT displayed fair overall internal reliability (0.607), slightly lower than 0.73 in Williams et al [12], and much lower than 0.85 in Mabiso et al [45] and 0.91 in Williams et al [13]. These differences may be attributed to the different sizes of samples in different studies on the one hand, 50 in our study, compared with 543 in Williams et al [12] and 161 in Mabiso et al [45], and to the different numbers of items on the scales in different studies on the other hand, 10 items on the scale in our study, in comparison with 30 items in Williams et al [12] and 16 items in Mabiso et al [45]. This finding supports the finding reported by Tavakol and Dennick [40] that *α* is influenced by the number of items on a test: more items on a test result in higher *α* values. The different internal consistency values of the B-CLAT when being administered among different populations point to the importance of developing and choosing B-CLATs that are linguistically and culturally appropriate to specific populations. As asserted by Williams et al [12], “A gap exists for linguistically and culturally sensitive measurements of functional breast cancer literacy that adheres to psychometric rigors.”

Williams et al [12] hypothesized and verified that family breast cancer history could lead to the highest scores on the B-CLAT. Our research results support this hypothesis. We found that no participants in our sample reported breast cancer history and they scored poorly on the C-B-CLAT, as evidenced by the deletion of 24 items to which the participants returned inconsistent responses in the test and the retest. The participants’ poor performance on these deleted items shows that these questionnaire items were too difficult for Chinese college students with no breast cancer history. After deleting these items, we found that Cronbach *α* for the 3 subscales and the entire scale increased to varying degrees, implying the improved validity of the C-B-CLAT.

The results of our study and some previous studies show the discrepancies in scoring performance on the original and translated B-CLAT scale among different ethnic groups and on different subscales of the instrument within the same ethnic group. Williams et al [12] ascertained that those with no breast cancer history had the highest scores on the subscale of prevention and control and the lowest scores on the subscale of awareness. Our results confirm this finding. Internal consistency for the awareness subscale was determined at 0.224 in our study, considerably lower than 0.57 (Black), 0.59 (Latina), and 0.52 (Arab) in Williams et al [12], and 0.53 in Mabiso et al [45]. Internal consistency for the screening and knowledge subscale was much better at 0.509 in our study, but still lower than 0.53 (Latina), 0.63 (Arab), and 0.71 (Black) in Williams et al [12], and 0.78 in Mabiso et al [45]. Internal consistency for the prevention and control subscale was determined at 0.730 in our study, slightly higher than 0.64 in Mabiso et al [45] and 0.69 (Black) in Williams et al [12], and considerably higher than 0.34 (Latina) and 0.40 (Arab) in Williams et al [12]. As with the internal consistency for the entire scale, the Cronbach *α* in our study (.607) was lower than .61 (Latina), .68 (Arab), .81 (Black), and .73 (total) in Williams et al [12], and lower than .85 in Mabiso et al [45]. These results show the varying levels of different aspects of breast cancer literacy among different populations. As such, it is imperative to deliver more targeted interventions and more effective education about the screening, prevention, and treatment of breast cancer among diverse populations.

This study addressed the need for health literacy measures to be rigorously tested [46]. The 3 subscales focus on different aspects essential for women to engage in breast cancer screening. For example, low scores on the screening and knowledge subscale may indicate that participants cannot tell the differences between screening modalities or they are not sure of the times of screening. This could ascertain the relevance of health literacy to breast cancer screening and inform the development of more targeted education interventions [47]. It was essential to have a tool-assessing skills for understanding information on breast cancer screening and services available and making appropriate decisions on screening [12].

### Implications

The results derived from the psychometric evaluation study of the Chinese version of the B-CLAT highlight the significance and need for an instrument to assess breast cancer literacy among Chinese populations. This study, therefore, addressed the need ascertained in existing literature for more health literacy scales, including breast cancer literacy instruments, which can be used to assess health literacy [8]. It is essential to have a tool measuring a woman’s possession of skills for understanding information on breast cancer screening and services available and being able to make appropriate screening decisions [12]. This is especially true in China, where the prevalence, death rate, and growth rate of cancer cases are relatively high compared to relevant figures reported for other countries [3-5]. The 3 subscales of the C-B-CLAT concentrate on 3 different aspects (ie, awareness, screening and knowledge, and prevention and control) essential for a woman to engage in breast cancer screening. For example, a woman scoring low on the screening and knowledge subscale may indicate that she cannot tell the differences between screening modalities or she is not aware how many times of screenings she needs to undergo. Drawing on such a scale specifically evaluating functional understanding of breast cancer, we can move “one step closer toward understanding the relation between this measure and breast cancer screening behaviors” [12]. We would, therefore, be informed of the relevance of health literacy to breast cancer screening and the need to design more targeted education interventions [47].

This study also addressed the need for health literacy scales to be rigorously tested [46]. An essential step in designing research interventions is to secure instruments that are linguistically and culturally appropriate [12]. As such, rigorous adaptation strategies need to be adopted to ensure the semantic equivalence and cultural relevance and appropriateness of the newly developed tools in the target language and culture. Validity and reliability testing can reveal the applicability and efficacy of health literacy instruments among specific target populations, which can inform the improvement of scales currently available and the development of new scales. As a result, the clinical screening of and health intervention in particular diseases could be made more effective and tailored using such rigorously tested tools. Validating instruments assessing functional health literacy specific to breast cancer makes a significant contribution to the science community and significantly influences the capacities of public health agencies to more effectively promote the screening and early detection of breast cancer [12].

### Limitations

Some limitations of this study need to be acknowledged. First, the relatively small size and the cohort nature of our sample may limit the generalizability of our research results and findings. We recruited a cohort of college students whose educational attainment was somewhat higher than that of the general Chinese women. Their age ranged from 17 to 22 years. As a result, we are not sure of the generalizability of our findings to other populations of different education levels and age groups. Future studies are warranted to verify the validity and reliability of the C-B-CLAT in large-sized samples with diverse educational attainments, age ranges, occupations, and ethnic groups. Second, it would also be necessary to validate the sensitivity of the newly developed instrument in diverse populations.

### Conclusions

In the context of cancer being the leading cause of death in China, a linguistically and culturally appropriate assessment tool is urgently needed to assess the cancer health literacy of Chinese-speaking populations. We translated and adapted the English version of the B-CLAT into a Chinese version (C-B-CLAT) and conducted a prospective psychometric evaluation study among 50 Chinese college students to verify its suitability for cancer literacy assessment. It had been proven valid and reliable for assessing cancer health literacy among this population.

## Abbreviations

B-CLAT: Breast Cancer Literacy Assessment Tool
C-B-CLAT: simplified-Chinese version of the B-CLAT
ICC: intraclass correlation coefficient
OR: odds ratio

## Data Availability

Data and materials are available via victorsyhz@hotmail.com upon request.

## References

[1] Ferlay J, Soerjomataram I, Dikshit R, Eser S, Mathers C, Rebelo M, Parkin DM, Forman D, Bray F (2015). Cancer incidence and mortality worldwide: sources, methods and major patterns in GLOBOCAN 2012. Int J Cancer.

[2] Fitzmaurice C, Dicker D, Pain A, Hamavid H, Moradi-Lakeh M, MacIntyre MF, Allen C, Hansen G, Woodbrook R, Wolfe C, Hamadeh RR, Moore A, Werdecker A, Gessner BD, Te Ao B, McMahon B, Karimkhani C, Yu C, Cooke GS, Schwebel DC, Carpenter DO, Pereira DM, Nash D, Kazi DS, De Leo D, Plass D, Ukwaja KN, Thurston GD, Yun Jin K, Simard EP, Mills E, Park EK, Catalá-López F, deVeber G, Gotay C, Khan G, Hosgood HD, Santos IS, Leasher JL, Singh J, Leigh J, Jonas JB, Sanabria J, Beardsley J, Jacobsen KH, Takahashi K, Franklin RC, Ronfani L, Montico M, Naldi L, Tonelli M, Geleijnse J, Petzold M, Shrime MG, Younis M, Yonemoto N, Breitborde N, Yip P, Pourmalek F, Lotufo PA, Esteghamati A, Hankey GJ, Ali R, Lunevicius R, Malekzadeh R, Dellavalle R, Weintraub R, Lucas R, Hay R, Rojas-Rueda D, Westerman R, Sepanlou SG, Nolte S, Patten S, Weichenthal S, Abera SF, Fereshtehnejad SM, Shiue I, Driscoll T, Vasankari T, Alsharif U, Rahimi-Movaghar V, Vlassov VV, Marcenes WS, Mekonnen W, Melaku YA, Yano Y, Artaman A, Campos I, MacLachlan J, Mueller U, Kim D, Trillini M, Eshrati B, Williams HC, Shibuya K, Dandona R, Murthy K, Cowie B, Amare AT, Antonio CA, Castañeda-Orjuela C, van Gool CH, Violante F, Oh IH, Deribe K, Soreide K, Knibbs L, Kereselidze M, Green M, Cardenas R, Roy N, Tillmann T, Li Y, Krueger H, Monasta L, Dey S, Sheikhbahaei S, Hafezi-Nejad N, Kumar GA, Sreeramareddy CT, Dandona L, Wang H, Vollset SE, Mokdad A, Salomon JA, Lozano R, Vos T, Forouzanfar M, Lopez A, Murray C, Naghavi M, Global Burden of Disease Cancer Collaboration (2015). The global burden of cancer 2013. JAMA Oncol.

[3] Jemal A, Bray F, Center MM, Ferlay J, Ward E, Forman D (2011). Global cancer statistics. CA Cancer J Clin.

[4] GLOBOCAN 2008: cancer incidence and mortality worldwide. IARC cancerbase No. 10. World Health Organization.

[5] Fan L, Strasser-Weippl K, Li JJ, St Louis J, Finkelstein DM, Yu KD, Chen WQ, Shao ZM, Goss PE (2014). Breast cancer in China. Lancet Oncol.

[6] Han H, Huh B, Kim MT, Kim J, Nguyen T (2014). Development and validation of the assessment of health literacy in breast and cervical cancer screening. J Health Commun.

[7] Lindau ST, Tomori C, McCarville MA, Bennett CL (2001). Improving rates of cervical cancer screening and Pap smear follow-up for low-income women with limited health literacy. Cancer Invest.

[8] Institute of Medicine (2004). Health Literacy: A Prescription to End Confusion.

[9] Weinick RM, Zuvekas SH, Cohen JW (2000). Racial and ethnic differences in access to and use of health care services, 1977 to 1996. Med Care Res Rev.

[10] Williams KP, Mullan PB, Fletcher F (2007). Working with African American women to develop a cancer literacy assessment tool. J Cancer Educ.

[11] Rivera-Vásquez O, Mabiso A, Hammad A, Williams KP (2009). A community-based approach to translating and testing cancer literacy assessment tools. J Cancer Educ.

[12] Williams KP, Templin TN, Hines RD (2013). Answering the call: a tool that measures functional breast cancer literacy. J Health Commun.

[13] Williams KP, Reckase M, Rivera-Vazquez O (2008). Toward the development of cancer literacy assessment tools. Michigan J Public Health.

[14] Dumenci L, Matsuyama R, Riddle DL, Cartwright LA, Perera RA, Chung H, Siminoff LA (2014). Measurement of cancer health literacy and identification of patients with limited cancer health literacy. J Health Commun.

[15] Porter P (2008). "Westernizing" women's risks? Breast cancer in lower-income countries. N Engl J Med.

[16] Anderson BO, Yip CH, Smith RA, Shyyan R, Sener SF, Eniu A, Carlson RW, Azavedo E, Harford J (2008). Guideline implementation for breast healthcare in low-income and middle-income countries: overview of the Breast Health Global Initiative Global Summit 2007. Cancer.

[17] Hortobagyi GN, de la Garza Salazar J, Pritchard K, Amadori D, Haidinger R, Hudis CA, Khaled H, Liu MC, Martin M, Namer M, O'Shaughnessy JA, Shen ZZ, Albain KS, ABREAST Investigators (2005). The global breast cancer burden: variations in epidemiology and survival. Clin Breast Cancer.

[18] Parkin DM (2001). Global cancer statistics in the year 2000. Lancet Oncol.

[19] Chang MC, Chen YC, Gau BS, Tzeng YF (2014). Translation and validation of an instrument for measuring the suitability of health educational materials in Taiwan: suitability assessment of materials. J Nurs Res.

[20] Zhao S, Cao Y, Cao H, Liu K, Lv X, Zhang J, Li Y, Davidson PM (2022). Chinese version of the mHealth app usability questionnaire: cross-cultural adaptation and validation. Front Psychol.

[21] Maneesriwongul W, Dixon JK (2004). Instrument translation process: a methods review. J Adv Nurs.

[22] Mohamad Marzuki MF, Yaacob NA, Yaacob NM (2018). Translation, cross-cultural adaptation, and validation of the Malay version of the system usability scale questionnaire for the assessment of mobile apps. JMIR Hum Factors.

[23] Epstein J, Santo RM, Guillemin F (2015). A review of guidelines for cross-cultural adaptation of questionnaires could not bring out a consensus. J Clin Epidemiol.

[24] Modiri O, Guha D, Alotaibi NM, Ibrahim GM, Lipsman N, Fallah A (2018). Readability and quality of wikipedia pages on neurosurgical topics. Clin Neurol Neurosurg.

[25] Streiner DL, Norman GR, Cairney J (2015). Health Measurement Scales: A Practical Guide to Their Development and Use. 5th ed.

[26] Wångdahl J, Jaensson M, Dahlberg K, Nilsson U (2020). The Swedish version of the electronic health literacy scale: prospective psychometric evaluation study including thresholds levels. JMIR Mhealth Uhealth.

[27] Barros A, Santos H, Moreira L, Santos-Silva F (2022). Translation and cross-cultural adaptation of the cancer health literacy test for Portuguese cancer patients: a pre-test. Int J Environ Res Public Health.

[28] Bourdieu P, Chamboredon JC, Passeron JC (1968). The Craft of Sociology: Epistemological Foundations.

[29] Wild D, Grove A, Martin M, Eremenco S, McElroy S, Verjee-Lorenz A, Erikson P, ISPOR Task Force for Translation and Cultural Adaptation (2005). Principles of good practice for the translation and cultural adaptation process for patient-reported outcomes (PRO) measures: report of the ISPOR Task force for translation and cultural adaptation. Value Health.

[30] Sperber AD (2004). Translation and validation of study instruments for cross-cultural research. Gastroenterology.

[31] Wang WL, Lee HL, Fetzer SJ (2006). Challenges and strategies of instrument translation. West J Nurs Res.

[32] Jones EG, Kay M (1992). Instrumentation in cross-cultural research. Nurs Res.

[33] Guillemin F, Bombardier C, Beaton D (1993). Cross-cultural adaptation of health-related quality of life measures: literature review and proposed guidelines. J Clin Epidemiol.

[34] Beaton DE, Bombardier C, Guillemin F, Ferraz MB (2000). Guidelines for the process of cross-cultural adaptation of self-report measures. Spine.

[35] Jääskeliänien R, Gambier Y, van Doorslaer L (2010). Think-aloud protocol. Handbook of Translation Studies.

[36] Zeugfang D, Wisetborisut A, Angkurawaranon C, Aramrattana A, Wensing M, Szecsenyi J, Krug K (2018). Translation and validation of the PACIC+ questionnaire: the Thai version. BMC Fam Pract.

[37] Doak CC, Doak LG, Root JH (1996). Teaching Patients With Low Literacy Skills. 2nd ed.

[38] Attribution 4.0 International (CC BY 4.0). Creative Commons.

[39] wenjuanxing.

[40] Tavakol M, Dennick R (2011). Making sense of Cronbach's alpha. Int J Med Educ.

[41] Weir JP (2005). Quantifying test-retest reliability using the intraclass correlation coefficient and the SEM. J Strength Cond Res.

[42] Oremus M, Oremus C, Hall GBC, McKinnon MC, ECT and Cognition systematic review team (2012). Inter-rater and test-retest reliability of quality assessments by novice student raters using the Jadad and Newcastle-Ottawa scales. BMJ Open.

[43] Giavarina D (2015). Understanding Bland Altman analysis. Biochem Med.

[44] Gravesande J, Richardson J, Griffith L, Scott F (2019). Test-retest reliability, internal consistency, construct validity and factor structure of a falls risk perception questionnaire in older adults with type 2 diabetes mellitus: a prospective cohort study. Arch Physiother.

[45] Mabiso A, Williams KP, Todem D, Templin TN (2010). Longitudinal analysis of domain-level breast cancer literacy among African-American women. Health Educ Res.

[46] Pleasant A, McKinney J (2011). Coming to consensus on health literacy measurement: an online discussion and consensus-gauging process. Nurs Outlook.

[47] Paasche-Orlow MK, Wolf MS (2010). Promoting health literacy research to reduce health disparities. J Health Commun.

